# Music participation, global cortical morphometry, and later language outcomes in the ABCD study

**DOI:** 10.3389/fpsyg.2026.1852932

**Published:** 2026-08-05

**Authors:** Avery Wang, Mekibib Altaye, Zhixiu Lu, Junqi Wang, Jiyo Athertya, Christine B. Chung, Jiang Du

**Affiliations:** 1 Department of Radiology, University of California, San Diego, San Diego, CA, United States; 2 The Seven Hills School, Cincinnati, OH, United States; 3 Division of Biostatistics and Epidemiology, Cincinnati Children’s Hospital Medical Center, Cincinnati, OH, United States; 4 Imaging Research Center, Cincinnati Children’s Hospital Medical Center, Cincinnati, OH, United States

**Keywords:** Adolescent Brain Cognitive Development (ABCD) Study, music participation, language development, cortical morphometry, magnetic resonance imaging, longitudinal cohort study

## Abstract

**Introduction:**

Associations between music participation and language outcomes in children have been widely reported, yet whether cortical morphometry statistically accounts for these relationships remains unclear, particularly at the population level.

**Methods:**

Using longitudinal data from the population-based Adolescent Brain Cognitive Development Study, we examined whether music participation at ages 9–10 years was associated with language outcomes at 2-year follow-up and whether global cortical morphometry statistically accounted for part of these associations. The analytic sample included 5,993 children, including a twin subsample of 936 children used for sensitivity analyses. Music participation was assessed using parent-reported measures of sustained participation and practice intensity. Language outcomes were assessed at 2-year follow-up using NIH Toolbox measures of picture vocabulary, oral reading recognition, and crystallized cognition. Candidate mediators included baseline global cortical surface area, total cortical volume, mean cortical thickness, and mean sulcal depth. Linear mixed-effects models and mediation analyses were adjusted for baseline language performance and a prespecified set of demographic, socioeconomic, prenatal/perinatal, behavioral, extracurricular, and site-related covariates.

**Results:**

Sustained music participation was associated with higher crystallized cognition, picture vocabulary, and oral reading recognition scores at 2-year follow-up, with adjusted standardized differences ranging from 0.08 to 0.17 standard deviation units. Global cortical surface area and volume, but not cortical thickness or sulcal depth, statistically accounted for 4.1%–8.1% of these associations. Each 1-standard-deviation increase in practice intensity was associated with slightly higher crystallized cognition and picture vocabulary scores, with adjusted standardized differences ranging from 0.04 to 0.06 standard deviation units, but was not associated with oral reading recognition. For the two significant outcomes, global cortical surface area and volume statistically accounted for 4.6%–6.5% of the observed associations. In the twin subsample, sustained music participation remained positively associated with all three language outcomes, whereas practice intensity was not significantly associated with any language outcome.

**Discussion:**

These findings provide longitudinal, population-based evidence that real-world music participation may be a relevant environmental correlate of language development in late childhood and that global cortical morphometry may statistically account for a small but measurable proportion of these associations.

## 1 Introduction

Music participation has been associated with language-related outcomes across childhood and early adolescence (ages 6–13), a developmental period during which language and literacy skills continue to develop rapidly. Evidence from observational studies, classroom-based and randomized interventions, and recent reviews and meta-analyses suggests that children who engage in music activities often perform better on measures of auditory-linguistic processing, phonological awareness, reading-related skills, and verbal abilities than peers without such experiences (Neves et al., 2022; Gordon et al., 2015; Pino et al., 2023; Slater et al., 2014; Moreno et al., 2009; Flaugnacco et al., 2015).

However, many studies in this literature have been conducted in relatively small samples or in specific educational and intervention settings, potentially limiting generalizability and increasing vulnerability to residual confounding. A meta-analysis of more than 50 intervention studies in children found that the apparent cognitive benefits of music training were modest and substantially attenuated in studies with more rigorous designs (Sala and Gobet, 2020). Associations between music training and language-related outcomes may partly reflect socioeconomic and familial factors, as well as pre-existing cognitive characteristics, and evidence from studies of children and adolescents suggests that correlations between music participation and verbal ability may not be entirely causal (Hille et al., 2011; Wesseldijk et al., 2023; Schellenberg and Lima, 2024). Consistent with this interpretation, a recent large-scale longitudinal analysis from the Adolescent Brain Cognitive Development (ABCD) Study reported modest prospective associations across several cognitive domains, including language-related measures, in relation to continuous music training over 2 years, with effects moderated by neighborhood socioeconomic deprivation (Habibi et al., 2025).

To contextualize how music participation may be associated with language-related outcomes, it is helpful to consider these associations within a broader framework of structural brain development. Prior research supports two relevant observations: musical training has been associated with differences in cortical morphology, and cortical morphometric variation has been associated with verbal, cognitive, and reading-related performance during childhood and adolescence. In children who begin instrumental training around ages 6 to 7, 15 months of lessons has been associated with structural changes in primary motor and auditory regions, alongside gains in musically relevant motor and auditory skills (Hyde et al., 2009). Further longitudinal work in school-age children (ages 6–11) has reported music-training-related macro- and microstructural brain changes (Habibi et al., 2018). A parallel body of work further suggests that cortical morphometry is relevant to language and broader cognitive development during childhood and adolescence. Across the age span of 6 to 18, cortical thickness has been linked to general cognitive performance (Karama et al., 2011), while developmental associations exist between verbal fluency and the structural organization of language-relevant cortical systems (Porter et al., 2011). In early adolescence, age-related morphometric refinement has been associated with better verbal learning and memory outcomes (Squeglia et al., 2013). Furthermore, longitudinal work following children from the prereading period through age 11 identified neuroanatomical precursors of later dyslexia, predominantly in primary sensory cortices, before formal reading instruction began (Clark et al., 2014).

Although early work often emphasized region-specific forms of training-related plasticity, broader reviews and observational studies suggest that musical practice is a complex, multimodal activity that engages distributed sensory, motor, and higher-order cognitive systems rather than a single focal brain region (Herholz and Zatorre, 2012). In youth samples, musical training has been associated with cortical maturation in regions implicated in motor planning and coordination, visuospatial processing, and emotion regulation (Hudziak et al., 2014). Although music and language rely on partly specialized neural circuits, global morphometric measures, including total cortical surface area and cortical volume, may serve as broad summary indices of cortical organization and neurodevelopment when relevant associations are distributed across multiple systems. This approach is consistent with large cohort studies showing that global or cortex-wide structural patterns are associated with neurobehavioral characteristics and cognition in children (Modabbernia et al., 2021; Patel et al., 2022).

These findings support the plausibility that broad structural brain development may partly account for reported associations between music participation and language-related outcomes. However, longitudinal statistical tests of this hypothesized broad brain-structure component in large, population-based pediatric cohorts remain limited, and prior reviews have emphasized ongoing uncertainty regarding the extent of transfer from music training to nonmusical outcomes (Sala and Gobet, 2017; Schellenberg and Lima, 2024). To address these gaps, the present study examined prospective associations between music participation at ages 9–10 years and language outcomes 2 years later and tested whether global cortical morphometry statistically accounted for part of these associations in a large longitudinal cohort (Casey et al., 2018). Global cortical morphometric measures were used as population-level summary indices of broad cortical structure, rather than as markers of localized neurobiological pathways. In this population-based cohort, music participation reflects real-world variation in engagement that differs in duration, intensity, type, and setting across children, capturing typical patterns of music involvement rather than intensive or highly specialized training programs. We further evaluated whether these associations persisted after adjustment for baseline language performance and a broad set of demographic, socioeconomic, prenatal/perinatal, behavioral, and extracurricular enrichment factors, and whether findings remained consistent in a twin subsample as a sensitivity analysis addressing shared familial influences. Because music participation is a common childhood activity, clarifying its associations with brain structure and language outcomes may inform understanding of environmental correlates of language development at the population level. We hypothesized that music participation would be associated with later language outcomes and that global cortical morphometry would statistically account for part of these associations.

## 2 Methods

### 2.1 Study population

This study used data from the Adolescent Brain Cognitive Development (ABCD) Study, data release 3.0, a longitudinal cohort of participants enrolled at ages 9–10 years across 21 U. S. sites (Garavan et al., 2018). All data were de-identified and available to qualified investigators through a data use agreement via the National Institute of Mental Health Data Archive. Follow-up assessments were conducted 2 years later, when participants were aged 11–12 years. Because of attrition and incomplete data, we initially identified a subset of participants (*n* = 6,571) with available 2-year follow-up data. To address potential attrition bias, we conducted an exploratory analysis of baseline characteristics to examine differential follow-up, informed by general guidance on selection bias in cohort studies (Nohr and Liew, 2018). Details of this exploratory analysis are provided in Supplementary eMethod 1. We found no evidence of systematic differences between participants with and without 2-year follow-up data, suggesting limited attrition-related selection bias.

**Figure 1:**
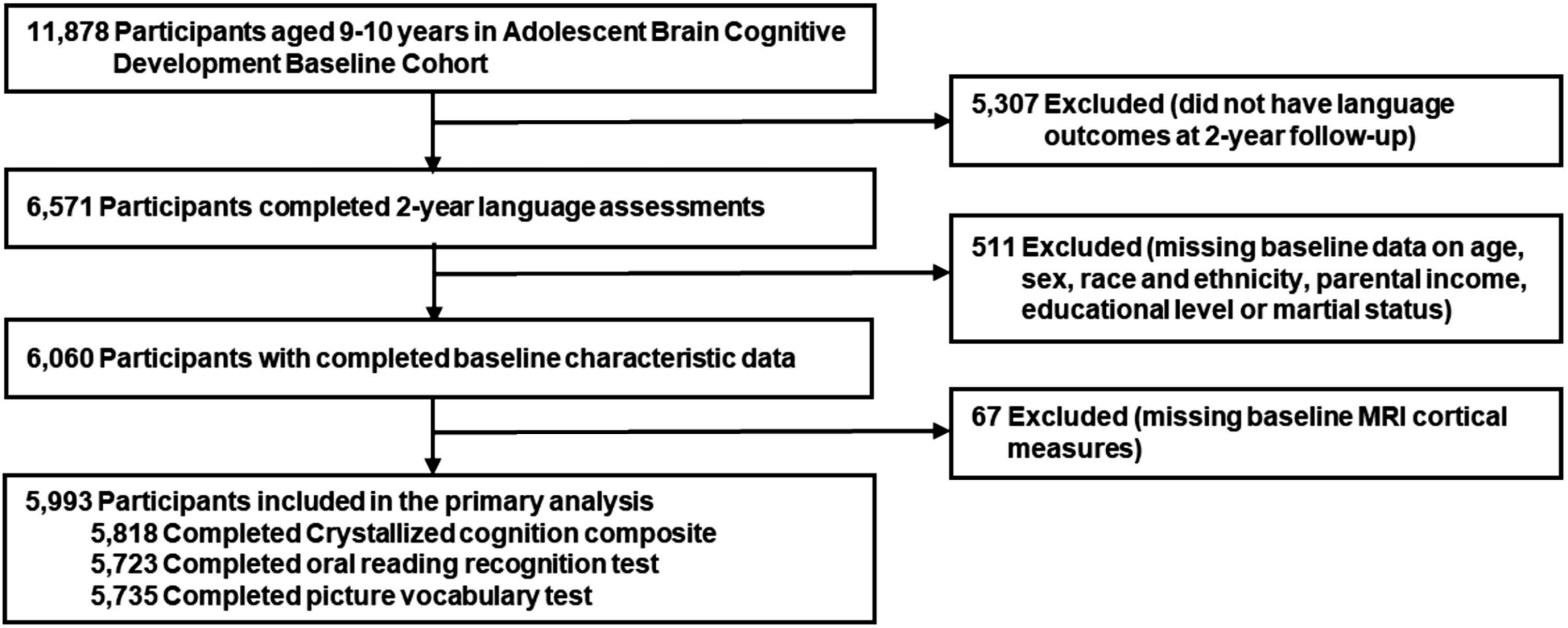
Flowchart of analytic sample selection.

The final analytic sample included 5,993 participants with complete data required for the primary analyses (Figure 1). A twin subsample (*n* = 936) nested within the overall analytic sample was also identified for sensitivity analyses to evaluate whether associations observed in the full cohort were also present among twins (Supplementary eFigure 1). The study was approved by the institutional review boards at all participating sites, with central IRB oversight by the University of California, San Diego. Parents or legal guardians provided written informed consent. The study followed the Strengthening the Reporting of Observational Studies in Epidemiology (STROBE) reporting guideline (von Elm et al., 2008). All methods were performed in accordance with applicable guidelines and regulations.

### 2.2 Measures

#### 2.2.1 Exposures: music participation (baseline)

Music participation measures were obtained from the parent-reported Sports and Activities Involvement Questionnaire (SAIQ) (Supplementary eTable 1). The primary exposure was sustained music participation, a binary variable defined as participation in organized music activities (e.g., singing, choir, guitar, piano, drums, violin, flute, band, rock band, orchestra) for at least 4 consecutive months (yes/no). This threshold (4 consecutive months) reflects regular, ongoing engagement, rather than brief or sporadic involvement, and was selected *a priori* based on the structure of the ABCD data collection rather than *post-hoc* optimization. A secondary exposure variable was practice intensity (minutes per week), modeled continuously in the full sample to capture weekly music exposure, with 0 indicating no reported music practice. Practice intensity was standardized to a *z*-score before analysis; coefficients therefore represent the association per 1-standard deviation (SD) increase in weekly practice intensity. Each exposure was analyzed in separate models to examine its association with baseline cortical structure and language outcomes assessed 2 years later. These measures reflect real-world extracurricular music participation in a population-based cohort.

#### 2.2.2 Outcomes: language assessments (2-year follow-up)

Language outcomes were assessed using the NIH Toolbox Cognition Battery (Gershon et al., 2014) (Supplementary eTable 2), which provides psychometrically validated measures of key language and reading abilities. The assessments included three primary outcomes: (1) the Picture Vocabulary Test, measuring receptive vocabulary and verbal comprehension; (2) the Oral Reading Recognition Test, assessing reading decoding and word recognition accuracy; and (3) the Crystallized Cognition Composite (Akshoomoff et al., 2013), an index of crystallized ability derived from the Picture Vocabulary and Oral Reading Recognition scores, reflecting accumulated knowledge and language-based skills. All scores were age-adjusted and standardized to the NIH Toolbox normative sample (mean = 100, SD = 15), with higher scores indicating better performance. These language measures have demonstrated strong reliability and validity in school-aged populations and are appropriate for assessing developmental variation in language-related skills (Gershon et al., 2014; Akshoomoff et al., 2013; Weintraub et al., 2013). The same NIH Toolbox language measures were administered at baseline and included as covariates in all longitudinal analyses to account for pre-existing differences in language ability, thereby supporting interpretation of the estimates as associations with follow-up language performance conditional on baseline ability.

#### 2.2.3 Mediators: global cortical measures (baseline)

Four baseline global cortical morphometric measures were examined as *a priori* potential mediators of the association between music participation and language outcomes: global cortical surface area, total cortical volume, mean cortical thickness, and mean sulcal depth (Supplementary eTable 3). Absolute global measures were selected as whole-cortex summary indices of cortical structure, consistent with prior developmental cohort studies (Modabbernia et al., 2021; Patel et al., 2022) and with methodological work showing that proportional intracranial volume correction can alter brain-behavior associations and introduce bias in analyses using cortical morphometric measures (Dhamala et al., 2022). However, these global measures are not designed to localize auditory, motor, or language-specific neural systems and should therefore be interpreted as summary markers rather than evidence of region-specific mechanisms. Detailed MRI acquisition, preprocessing, and quality control procedures are described in Supplementary eMethod 2.

#### 2.2.4 Covariates

Analyses were adjusted for prespecified covariates selected *a priori* based on prior literature, plausible confounding structure, and established associations with music participation and developmental outcomes. Covariates were grouped into five conceptual domains (Supplementary eTable 4): demographic factors (child age, sex, and parent-reported race and ethnicity), socioeconomic indicators (parental income, education, and marital status) (Rakesh et al., 2022; Miksza, 2007), prenatal/perinatal factors (preterm birth and maternal tobacco use during pregnancy) (Kelly et al., 2024; El Marroun et al., 2014), child behavioral and emotional functioning assessed using Child Behavior Checklist (CBCL/6–18) *T*-scores for Attention-Deficit/Hyperactivity Disorder (ADHD) Problems based on DSM-5 criteria, Social Problems, and broadband Internalizing and Externalizing domains (Achenbach and Rescorla, 2001), and participation in non-music extracurricular activities (sports and creative arts), modeled as the total count of activities as a proxy for overall extracurricular engagement and environmental enrichment (Crosnoe et al., 2015). To reduce overadjustment and multicollinearity among correlated prenatal/perinatal and behavioral variables, representative variables from these domains were included in the primary models, whereas models including additional variables from these domains were conducted as sensitivity analyses. The primary covariate set included age, sex, race and ethnicity, parental income and education, marital status, maternal tobacco use during pregnancy, CBCL ADHD *T*-score, and number of non-music extracurricular activities. All models additionally adjusted for baseline language performance and study site to account for pre-existing differences in language ability and for site-related variation in recruitment, data collection, and imaging procedures (Casey et al., 2018; Garavan et al., 2018).

### 2.3 Statistical analysis

Baseline sample characteristics were summarized using means (SD) for continuous variables and proportions for categorical variables. Standardized mean differences (SMDs) were calculated to compare characteristics between (1) the full analysis sample and the twin subsample and (2) participants with and without sustained music participation. SMDs were computed using Cohen’s *d* for continuous variables and Cohen’s *h* for categorical variables (Cohen, 1988). Pearson correlation coefficients were calculated between each baseline characteristic and the continuous music exposure variable (practice intensity) (Rodgers and Nicewander, 1988).

#### 2.3.1 Statistical mediation analyses

**Figure 2:**
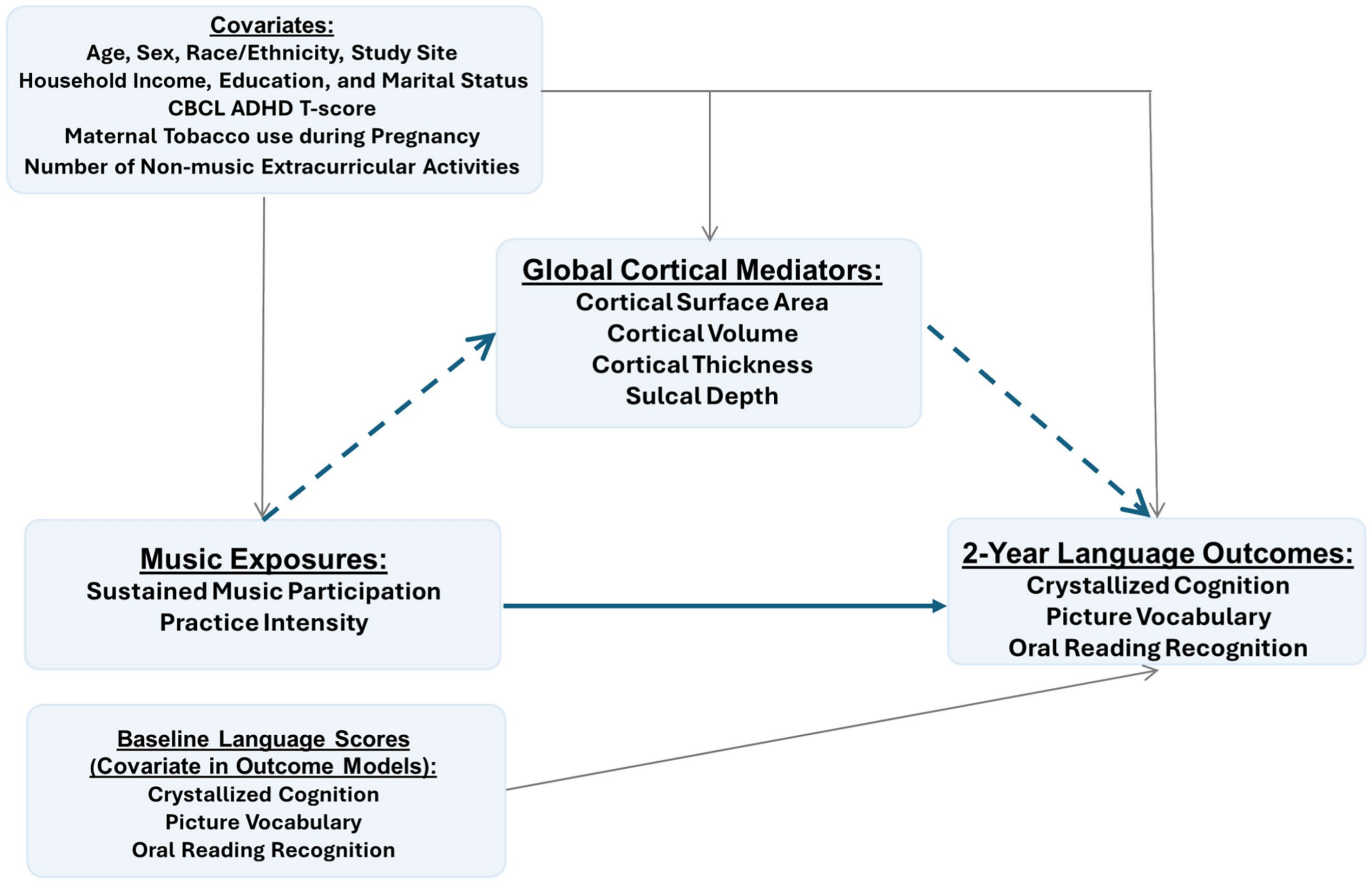
Conceptual model illustrating hypothesized associations between music participation, global cortical morphometric measures, and language outcomes at 2-year follow-up. Arrows denote hypothesized statistical associations used to guide model specification and covariate adjustment and do not imply causal effects. The solid blue arrow indicates the association between music participation and language outcomes. Dashed blue arrows indicate the mediated pathway through global cortical morphometric measures. Solid gray arrows indicate covariates included for statistical adjustment in the corresponding models. Baseline language scores were included as covariates in the outcome models to account for baseline differences in language ability and to support longitudinal interpretation.

We used mediation models as an associational decomposition to evaluate whether global cortical morphometric measures were associated with both music participation and subsequent language outcomes, and whether these measures statistically accounted for part of the prospective association between music participation and language outcomes assessed 2 years later (Figure 2). We defined two baseline exposures (sustained music participation, practice intensity), four baseline mediators (cortical surface area, cortical volume, cortical thickness, and sulcal depth), and three outcomes at 2-year follow-up (picture vocabulary, oral reading recognition, and crystallized cognition composite). This design yielded 24 exposure-mediator-outcome combinations (2 × 4 × 3). Each exposure-mediator-outcome combination was modeled separately to reflect conceptual distinctions among constructs and to minimize collinearity.

For each combination, we fit three linear mixed-effects models (LMMs) (Pinheiro and Bates, 2000) corresponding to the standard statistical mediation framework (MacKinnon et al., 2007): (1) an outcome-exposure model estimating the total effect of the exposure on the follow-up outcome, without including the mediator (Equation 1); (2) a mediator-exposure model estimating the association between the exposure and the mediator (Equation 2); and (3) an outcome-mediator-exposure model estimating the association between the mediator and the outcome while adjusting for the exposure, yielding the direct-effect estimate (Equation 3).


$$Y_{\mathrm{ij}(t=2)}=\beta_{T}X_{\mathrm{ij}(t=0)}+\Gamma_{1}C_{\mathrm{ij}}+\gamma_{1}S_{i}+\delta_{1}Y_{\mathrm{ij}(t=0)}+u_{j}+\varepsilon_{\mathrm{ij}}$$ (1)

$$M_{\mathrm{ij}(t=0)}=\alpha X_{\mathrm{ij}(t=0)}+\Gamma_{2}C_{\mathrm{ij}}+\gamma_{2}S_{i}+u_{j}+\varepsilon_{\mathrm{ij}}$$ (2)

$$\begin{array}{l} Y_{\mathrm{ij}(t=2)}=\beta_{D}X_{\mathrm{ij}(t=0)}+\beta_{M}M_{\mathrm{ij}(t=0)}+\Gamma_{3}C_{\mathrm{ij}}+\gamma_{3}S_{i}\\ +\delta_{2}Y_{\mathrm{ij}(t=0)}+u_{j}+\varepsilon_{\mathrm{ij}} \end{array}$$ (3)

Let i index the individual and j index the family unit. $Y_{\mathrm{ij}(t=0)}$ and $Y_{\mathrm{ij}(t=2)}$ denote the language score at baseline and 2-year follow-up, respectively; $X_{\mathrm{ij}(t=0)}$ denotes the baseline exposure, $M_{\mathrm{ij}(t=0)}$ denotes the baseline mediator, $C_{\mathrm{ij}}$ denotes the vector of predefined covariates, $S_{i}$ denotes study site, $u_{j}$ denotes the random intercept for family ID, and $\varepsilon_{\mathrm{ij}}$ denotes the error term. *Γ*₁, Γ₂, and Γ₃ denote coefficient vectors for covariates, *γ*₁, γ₂, and γ₃ denote fixed-effect coefficients for study site; and δ₁ and δ₂ denote coefficients for baseline language performance in the total and direct effect models, respectively. $\beta_{T}$ denotes the total-effect parameter, $\beta_{D}$ denotes the direct-effect parameter conditional on the mediator, $\beta_{M}$ denotes the association between the mediator and the follow-up outcome after adjusting for the exposure and covariates, and α denotes the association between the exposure and the mediator. The indirect-effect parameter was calculated as $\alpha \times \beta_{M}$ . The proportion of the total association statistically accounted for by the mediator was calculated as $\alpha \beta_{M}/\beta_{T}$ . To aid interpretation of effect size, unstandardized total-effect estimates $\beta_{T}$ for NIH Toolbox language outcomes were expressed in SD units by dividing each estimate by the NIH Toolbox normative SD of 15. For sustained music participation, the resulting values approximate adjusted standardized mean differences; for practice intensity, they represent standardized outcome differences per 1-SD increase in weekly practice intensity. Baseline language performance was included as a covariate in the total and direct effect models so that estimates represent associations with follow-up language outcomes conditional on baseline performance. Because the exposure and mediator were both measured at baseline, whereas language outcomes were measured at follow-up, this mediation framework evaluates whether baseline brain structure statistically accounts for part of the association between baseline music participation and later language outcomes, but it does not establish temporal ordering between exposure and mediator. Accordingly, we retained standard mediation model terms such as indirect effect to describe model parameters, but interpreted these estimates as associational rather than causal. Ninety-five percent confidence intervals for indirect effects were estimated using Monte Carlo simulation (Preacher and Selig, 2012), and confidence intervals for total and direct effects were calculated using Wald-type methods. Multiple comparisons were addressed using the Benjamini-Hochberg false discovery rate (FDR) procedure (Benjamini and Hochberg, 1995), with a two-sided FDR-adjusted *q* < 0.05 considered statistically significant. All analyses were conducted in Python 3.11 using the *statsmodels* library (Seabold and Perktold, 2010).

#### 2.3.2 Twin sensitivity analysis

Twin analyses provided a sensitivity analysis of whether associations observed in the full cohort were also present in the twin subsample. Mediation models were repeated in the twin subsample using the same specification as in the full sample (Equations 1–3) and the same covariate adjustments. Family ID was included as a random intercept to account for within-family clustering, and zygosity (monozygotic vs. dizygotic) was included as a fixed effect to adjust for differences between twin types (Peper et al., 2009). This approach accounts for the non-independence of twin observations, but it does not estimate co-twin within-pair effects or fully control for shared familial confounding.

## 3 Results

### 3.1 Study population

Among 5,993 participants (baseline mean age, 10.0 [SD, 0.6] years; 47.1% female), the sample included 123 (2.1%) Asian, 650 (10.8%) Black, 1,098 (18.3%) Hispanic, and 3,530 (58.9%) White participants, as well as 592 (9.9%) participants identifying as multiracial and/or other races. The analytic twin subsample included 936 participants. Table 1 summarizes baseline characteristics of the full analytic sample and the twin subsample. Most demographic, behavioral, and extracurricular characteristics were similar between groups (absolute SMDs < 0.20). However, the twin subsample had a higher prevalence of preterm birth (57.0% vs. 19.9%; SMD, 0.79), a higher proportion of participants from households with annual income ≥$50,000 (93.3% vs. 73.4%; SMD, 0.56), and a lower proportion of Hispanic participants (8.4% vs. 18.3%; SMD, 0.30). Because the twin subsample differed from the full cohort in several baseline characteristics, the twin analyses were interpreted as sensitivity analyses rather than direct population-level estimates.

**Table 1:** Baseline characteristics of the full analytic sample and twin subsample.

Characteristic	Full sample (*n* = 5,993)	Twin subsample (*n* = 936)	SMD^a^
Demographics			
Age, y	10.0 (0.6)	10.2 (0.6)	−0.36
Sex			
Female	2,823 (47.1)	454 (48.5)	−0.03
Male	3,170 (52.9)	482 (51.5)	0.03
Race and ethnicity			
Asian	123 (2.1)	1 (0.1)	0.23
Black	650 (10.8)	122 (13.0)	−0.07
Hispanic	1,098 (18.3)	79 (8.4)	0.30
White	3,530 (58.9)	666 (71.2)	−0.26
Multiracial and/or others^b^	592 (9.9)	68 (7.3)	0.09
Socioeconomic factors			
Annual household income, $			
<50,000	1,594 (26.6)	63 (6.7)	0.56
≥50,000	4,399 (73.4)	873 (93.3)	−0.56
Parental marital status			
Married	4,296 (71.7)	716 (76.5)	−0.11
Living with partner	283 (4.7)	18 (1.9)	0.16
Single	1,414 (23.6)	202 (21.6)	0.05
Parental education			
< Some college	757 (12.6)	63 (6.7)	0.20
≥ Some college	5,236 (87.4)	873 (93.3)	−0.20
Prenatal and perinatal factors			
Parent-reported preterm-birth status			
Yes	1,191 (19.9)	534 (57.0)	−0.79
No	4,736 (79.0)	394 (42.1)	0.77
Do not know	66 (1.1)	8 (0.9)	0.02
Maternal alcohol use in pregnancy			
Yes	178 (3.0)	17 (1.8)	0.08
No	5,677 (94.7)	913 (97.5)	−0.15
Do not know	138 (2.3)	6 (0.6)	0.15
Maternal tobacco use in pregnancy			
Yes	251 (4.2)	39 (4.2)	0.00
No	5,605 (93.5)	891 (95.2)	−0.07
Do not know	137 (2.3)	6 (0.6)	0.15
Child behavior checklist (CBCL)^c^			
ADHD	53.1 (5.6)	52.4 (4.9)	0.13
Social	52.7 (4.5)	52.0 (3.6)	0.17
Internalizing	48.6 (10.5)	46.7 (10.2)	0.18
Externalizing	45.5 (10.2)	44.1 (9.9)	0.14
Broader enrichment activities			
Non-music activities^d^	2.83 (2.01)	2.91 (2.00)	−0.04

Values are mean [standard deviation (SD)] for continuous variables or No. (%) for categorical variables.

a Standardized mean difference (SMD) was calculated using Cohen’s d for continuous variables and Cohen’s h for categorical variables. Positive values indicate higher means or proportions in the full analytic sample relative to the twin subsample.

b Primary caregivers could select multiple racial or ethnic subgroups for their children; the multiracial and/or others category includes participants with multiple reported categories or no specific race or ethnicity identified.

c ADHD, Social, Internalizing, and Externalizing refer to Child Behavior Checklist (CBCL) *T*-scores, standardized to a mean of 50 (SD, 10).

d Non-music activities were defined as the total count of non-music extracurricular activity items, including team sports, individual sports, and creative arts, in which the child participated for ≥4 continuous months, as reported in the Sports and Activities Involvement Questionnaire.

### 3.2 Baseline characteristics by music exposure variables

At baseline, 2,558 (42.7%) participants engaged in sustained music participation (Table 2). Participants with sustained music participation were more likely to come from higher-income households (≥$50,000: 85.0% vs. 64.8%; SMD, 0.48), have parents with higher educational attainment (≥some college: 94.0% vs. 82.4%; SMD, 0.37), and have married parents (81.5% vs. 64.4%; SMD, 0.39). They also participated more frequently in other non-music extracurricular activities (mean [SD], 3.58 [2.09] vs. 2.27 [1.75]; SMD, 0.69). These differences indicate that sustained music participation was associated with socioeconomic and enrichment-related factors, consistent with the possibility of confounding. Differences in other baseline characteristics were generally smaller in magnitude. Baseline correlations for practice intensity, modeled in the full sample with 0 indicating no reported music practice, showed a similar overall pattern and are presented in Supplementary eTable 5.

**Table 2:** Baseline characteristics by sustained music participation status in the full sample.

Characteristic	Sustained music participation^a^ (*n* = 2,558)	No sustained music participation (*n* = 3,435)	SMD^b^
Demographics			
Age, y	10.0 (0.6)	9.89 (0.61)	0.18
Sex			
Female	1,344 (52.5)	1,479 (43.1)	0.19
Male	1,214 (47.5)	1,956 (56.9)	−0.19
Race and ethnicity			
Asian	75 (2.9)	48 (1.4)	0.10
Black	192 (7.5)	458 (13.3)	−0.19
Hispanic	351 (13.7)	747 (21.7)	−0.21
White	1,704 (66.6)	1,826 (53.2)	0.28
Multiracial and/or others^c^	236 (9.2)	356 (10.4)	−0.04
Socioeconomic factors			
Annual household income			
<50,000	383 (15.0)	1,211 (35.2)	−0.48
≥50,000	2,175 (85.0)	2,224 (64.8)	0.48
Parental marital status			
Married	2,084 (81.5)	2,212 (64.4)	0.39
Living with partner	58 (2.3)	225 (6.6)	−0.21
Single	416 (16.3)	998 (29.1)	−0.31
Parental education			
< Some college	154 (6.0)	603 (17.6)	−0.37
≥ Some college	2,404 (94.0)	2,832 (82.4)	0.37
Prenatal and perinatal factors			
Parent-reported preterm-birth status			
Yes	448 (17.5)	743 (21.6)	−0.10
No	2,083 (81.4)	2,653 (77.2)	0.10
Do not know	27 (1.1)	39 (1.1)	0.00
Maternal alcohol use in pregnancy			
Yes	96 (3.8)	82 (2.4)	0.08
No	2,405 (94.0)	3,272 (95.3)	−0.06
Do not know	57 (2.2)	81 (2.4)	−0.01
Maternal tobacco use in pregnancy			
Yes	57 (2.2)	194 (5.6)	−0.18
No	2,448 (95.7)	3,157 (91.9)	0.16
Do not know	53 (2.1)	84 (2.4)	−0.02
Child Behavior Checklist (CBCL)^d^			
ADHD	52.7 (5.2)	53.4 (5.8)	−0.13
Social	52.3 (4.2)	52.9 (4.8)	−0.13
Internalizing	48.3 (10.3)	48.8 (10.6)	−0.05
Externalizing	44.6 (9.7)	46.2 (10.5)	−0.16
Broader enrichment activities			
Non-music activities^e^	3.58 (2.09)	2.27 (1.75)	0.69

Values are mean (SD) for continuous variables or No. (%) for categorical variables.

a Sustained music participation was defined as participation in organized music activities for ≥4 continuous months based on parent report in the Sports and Activities Involvement Questionnaire.

b Standardized mean difference (SMD) was calculated using Cohen’s *d* for continuous variables and Cohen’s h for categorical variables. Positive values indicate higher means or proportions in the sustained music participation group.

c Primary caregivers could select multiple racial or ethnic subgroups for their children; the multiracial and/or others category includes participants with multiple reported categories or no specific race or ethnicity identified.

d ADHD, Social, Internalizing, and Externalizing refer to Child Behavior Checklist (CBCL) *T*-scores, standardized to a mean of 50 (SD, 10).

e Non-music activities were defined as the total count of non-music extracurricular activity items, including team sports, individual sports, and creative arts, in which the child participated for ≥4 continuous months, as reported in the Sports and Activities Involvement Questionnaire.

### 3.3 Global cortical morphometry in longitudinal associations between music participation and language outcomes

#### 3.3.1 Primary exposure: sustained music participation

In models adjusted for baseline language performance and the primary covariate set, sustained music participation at baseline was positively associated with language outcomes assessed at 2-year follow-up, including crystallized cognition (*β* = 2.11; 95% CI, 1.43 to 2.80), picture vocabulary (*β* = 2.50; 95% CI, 1.75 to 3.26), and oral reading recognition (*β* = 1.24; 95% CI, 0.54 to 1.94) (Table 3). Relative to the NIH Toolbox normative SD of 15, the total-effect estimates corresponded to adjusted standardized differences in language outcomes of approximately 0.14, 0.17, and 0.08 SD units, respectively. Indirect-effect estimates were statistically significant for global cortical surface area and volume, but not for cortical thickness or sulcal depth. In the primary models, global cortical surface area and volume statistically accounted for approximately 4.1 to 8.1% of the total associations across outcomes. In models additionally adjusted for expanded prenatal/perinatal and behavioral variables, total and indirect-effect estimates were modestly attenuated, but the overall pattern of findings remained similar: sustained music participation remained significantly associated with all language outcomes, indirect-effect estimates for global cortical surface area and volume remained statistically significant, and the proportion of the association accounted for by each measure was slightly smaller in the expanded models (Supplementary eTable 6).

**Table 3:** Global cortical morphometric measures as candidate mediators for the associations between baseline music exposure variables and language outcomes at 2-year follow-up in the full analytic sample.

Baseline exposure	2-year language outcome	Cortical mediator	Total effect, $\beta_{T}\;$ (95% CI)	Indirect effect $\;\beta_{I}\;$ (95% CI)	*q*-value for $\beta_{I}$	Direct effect $\beta_{D}$ (95% CI)	Proportion mediated, %
Sustained music participation	Crystallized cognition composite (*N* = 5,818)	Thickness	2.114 (1.427, 2.802)	0.001 (−0.019, 0.022)	0.934	2.114 (1.426, 2.801)	—
		Sulcal depth		−0.001 (−0.015, 0.012)	0.934	2.115 (1.427, 2.802)	—
		Surface area		0.111 (0.038, 0.194)	0.005	2.049 (1.366, 2.732)	5.3
		Volume		0.117 (0.040, 0.203)	0.005	2.045 (1.362, 2.728)	5.5
	Picture vocabulary test (*N* = 5,735)	Thickness	2.504 (1.752, 3.256)	0.000 (−0.013, 0.014)	0.935	2.504 (1.752, 3.256)	—
		Sulcal depth		−0.002 (−0.023, 0.017)	0.935	2.504 (1.752, 3.256)	—
		Surface area		0.108 (0.036, 0.191)	0.006	2.441 (1.693, 3.190)	4.3
		Volume		0.102 (0.034, 0.183)	0.006	2.445 (1.696, 3.194)	4.1
	Oral reading recognition test (*N* = 5,723)	Thickness	1.238 (0.542, 1.935)	0.001 (−0.027, 0.030)	0.934	1.237 (0.540, 1.933)	—
		Sulcal depth		0.000 (−0.010, 0.012)	0.934	1.239 (0.542, 1.935)	—
		Surface area		0.084 (0.028, 0.152)	0.007	1.187 (0.493, 1.881)	6.8
		Volume		0.100 (0.034, 0.178)	0.007	1.177 (0.484, 1.870)	8.1
Practice intensity	Crystallized cognition composite (*N* = 5,818)	Thickness	0.665 (0.357, 0.973)	0.000 (−0.009, 0.010)	0.921	0.665 (0.357, 0.974)	—
		Sulcal depth		0.001 (−0.005, 0.008)	0.921	0.664 (0.356, 0.973)	—
		Surface area		0.039 (0.006, 0.075)	0.026	0.637 (0.330, 0.943)	5.9
		Volume		0.043 (0.009, 0.082)	0.026	0.636 (0.330, 0.943)	6.5
	Picture vocabulary test (*N* = 5,735)	Thickness	0.834 (0.494, 1.175)	0.000 (−0.006, 0.006)	0.922	0.834 (0.493, 1.175)	—
		Sulcal depth		0.001 (−0.007, 0.011)	0.922	0.833 (0.492, 1.174)	—
		Surface area		0.038 (0.006, 0.074)	0.027	0.810 (0.471, 1.150)	4.6
		Volume		0.038 (0.008, 0.073)	0.027	0.810 (0.471, 1.149)	4.6
	Oral reading recognition test (*N* = 5,723)	Thickness	0.279 (−0.038, 0.595)	0.001 (−0.012, 0.014)	0.921	0.279 (−0.038, 0.595)	—
		Sulcal depth		0.000 (−0.006, 0.005)	0.921	0.279 (−0.038, 0.595)	—
		Surface area		0.029 (0.004, 0.059)	0.030	0.261 (−0.054, 0.577)	—
		Volume		0.037 (0.007, 0.071)	0.030	0.258 (−0.057, 0.573)	—

Models were adjusted for baseline language performance; demographic factors (age, sex, and race and ethnicity); socioeconomic factors (household income, parental education, and marital status); maternal tobacco use during pregnancy; CBCL ADHD *T*-score; number of non-music extracurricular activities (sports and creative arts); and study site. $\beta_{T}$ denotes the total effect, $\beta_{D}$ denotes the direct effect adjusted for the mediator, and $\beta_{I}\;$ denotes the indirect effect. The indirect effect was estimated using the product-of-coefficients method; the proportion mediated was calculated as the indirect effect divided by the total effect. Ninety-five percent confidence intervals for indirect effects were estimated using Monte Carlo simulation, whereas confidence intervals for total and direct effects were calculated using Wald-type methods. *q* values are *p-*values adjusted for multiple comparisons using the Benjamini-Hochberg false discovery rate procedure; *q* < 0.05 was considered statistically significant. The total effect reflects the association between the exposure and outcome and is therefore identical across mediator-specific models. “—” indicates that the proportion mediated was not estimated because either the indirect effect or the total effect was not statistically significant.

#### 3.3.2 Secondary exposure: practice intensity

In the full sample, each 1-SD increase in weekly practice intensity, greater practice intensity was positively associated with crystallized cognition (*β* = 0.67; 95% CI, 0.36 to 0.97) and picture vocabulary (*β* = 0.83; 95% CI, 0.49 to 1.18), but not oral reading recognition (*β* = 0.28; 95% CI, −0.04 to 0.60). These total-effect estimates corresponded to adjusted standardized differences in language outcomes of approximately 0.04, 0.06, and 0.02 SD units, respectively. For crystallized cognition and picture vocabulary, indirect-effect estimates were statistically significant for global cortical surface area and volume, but not for cortical thickness or sulcal depth. For crystallized cognition, global cortical surface area and volume statistically accounted for 5.9 and 6.5% of the total association, respectively. For picture vocabulary, global cortical surface area and volume each statistically accounted for 4.6% of the total association. Although indirect-effect estimates for global cortical surface area and volume were also statistically significant for oral reading recognition, the absence of a statistically significant total effect limits interpretation of this finding.

### 3.4 Twin sensitivity analyses

Sensitivity analyses were conducted in the twin subsample (Table 4). Sustained music participation remained positively associated with crystallized cognition (*β* = 2.50; 95% CI, 0.70 to 4.31), picture vocabulary (*β* = 2.79; 95% CI, 0.82 to 4.76), and oral reading recognition (*β* = 1.95; 95% CI, 0.20 to 3.71). These total-effect estimates corresponded to adjusted standardized differences of approximately 0.17, 0.19, and 0.13 SD units, respectively. Indirect-effect estimates for global cortical surface area and volume were statistically significant, statistically accounting for approximately 10.2 to 14.0% of the total associations across outcomes. Cortical thickness and sulcal depth did not show statistically significant indirect-effect estimates in the twin analyses. In the twin subsample, analyses using practice intensity did not demonstrate statistically significant associations with language outcomes or statistically significant indirect-effect estimates for any cortical morphometric measure.

**Table 4:** Global cortical morphometric measures as candidate mediators of the associations between baseline sustained music participation and language outcomes at 2-year follow-up in the twin subsample.

Baseline exposure	2-year language outcome	Cortical mediator	Total effect, $\beta_{T}\;$ (95% CI)	Indirect effect $\;\beta_{I}\;$ (95% CI)	*q*-value for $\beta_{I}$	Direct effect $\beta_{D}$ (95% CI)	Proportion mediated, %
Sustained music participation	Crystallized cognition composite (*N* = 913)	Thickness	2.504 (0.697, 4.311)	0.034 (−0.070, 0.182)	0.795	2.462 (0.655, 4.270)	—
		Sulcal depth		−0.005 (−0.090, 0.066)	0.795	2.513 (0.704, 4.323)	—
		Surface area		0.329 (0.087, 0.658)	0.021	2.245 (0.45, 4.040)	13.1
		Volume		0.345 (0.090, 0.687)	0.021	2.215 (0.423, 4.008)	13.8
	Picture vocabulary test (*N* = 909)	Thickness	2.787 (0.817, 4.756)	0.028 (−0.067, 0.170)	0.616	2.754 (0.784, 4.724)	—
		Sulcal depth		−0.019 (−0.140, 0.067)	0.616	2.827 (0.855, 4.798)	—
		Surface area		0.283 (0.055, 0.604)	0.038	2.569 (0.604, 4.533)	10.2
		Volume		0.285 (0.057, 0.610)	0.038	2.553 (0.590, 4.517)	10.2
	Oral reading recognition test (*N* = 908)	Thickness	1.952 (0.200, 3.705)	0.015 (−0.062, 0.123)	0.644	1.931 (0.176, 3.686)	—
		Sulcal depth		0.014 (−0.063, 0.121)	0.644	1.932 (0.180, 3.685)	—
		Surface area		0.272 (0.059, 0.572)	0.034	1.735 (−0.009, 3.479)	13.9
		Volume		0.274 (0.059, 0.577)	0.034	1.714 (−0.031, 3.458)	14.0

Results for practice intensity are not shown because no statistically significant total or indirect effects were observed for any language outcome in the twin subsample.

Models were adjusted for baseline language performance; demographic factors (age, sex, and race and ethnicity); socioeconomic factors (household income, parental education, and marital status); maternal tobacco use during pregnancy; CBCL ADHD *T*-score; number of non-music extracurricular activities (sports and creative arts); study site; and zygosity as a fixed effect, with family ID included as a random intercept. *β_T* denotes the total effect, *β_D* denotes the direct effect adjusted for the mediator, and *β_I* denotes the indirect effect. The indirect effect was estimated using the product-of-coefficients method, and the proportion mediated was calculated as the indirect effect divided by the total effect. Ninety-five percent confidence intervals for indirect effects were estimated using Monte Carlo simulation, whereas confidence intervals for total and direct effects were calculated using Wald-type methods. *q* values are *p-*values adjusted for multiple comparisons using the Benjamini-Hochberg false discovery rate procedure; *q* < 0.05 was considered statistically significant. The total effect reflects the association between the exposure and outcome and is therefore identical across mediator-specific models. “—” indicates that the proportion mediated was not estimated because either the indirect effect or the total effect was not statistically significant.

## 4 Discussion

In this large, population-based longitudinal cohort, sustained music participation in late childhood at ages 9 to 10 years was associated with modestly higher language performance 2 years later across crystallized cognition, picture vocabulary, and oral reading recognition. Mediation analyses indicated that global cortical surface area and volume statistically accounted for a small portion of these associations. These analyses adjusted for baseline language performance and a prespecified set of demographic, socioeconomic, prenatal/perinatal, behavioral, and extracurricular covariates.

### 4.1 Associations between music participation and language performance

One plausible explanation for the observed association between music participation and higher scores on picture vocabulary, oral reading recognition, and crystallized cognition is enhanced auditory processing. Both music and language engage partially overlapping neural circuits involved in processing sound, recognizing rhythmic patterns, and tracking sequential information (Patel, 2011; Peretz and Zatorre, 2005). Music participation requires attention to subtle variations in pitch, rhythm, and timbre, which may promote greater sensitivity to fine auditory detail and thereby support phonological awareness, the ability to recognize and manipulate sounds within words (Goswami, 2015). Phonological awareness is an important component of language development and is relevant to reading and speech processing. Through regular engagement with music, children may strengthen auditory discrimination and temporal processing in ways that support language-related tasks (Chobert et al., 2014; Besson et al., 2011).

Music participation may also be associated with processes beyond auditory perception, including attention, working memory, and cognitive flexibility (Besson et al., 2011; Moreno et al., 2011). These processes may help children maintain and manipulate information, monitor sequential patterns, and adapt to changing task demands, all of which are relevant to language learning and reading. Music practice often involves sustained attention, sequential monitoring, repetition, and feedback, which may help strengthen cognitive skills that also support language comprehension and use. However, the present study was not designed to directly test these behavioral mechanisms.

The more robust findings for sustained music participation than for practice intensity may suggest that ongoing involvement in music is a more stable and informative indicator of real-world engagement than weekly practice minutes in this population-based cohort. Sustained participation may better capture continued exposure to structured musical experiences over time, whereas practice intensity may be more variable, more vulnerable to measurement error, and more influenced by family routines or access-related factors. Because practice intensity was modeled in the full sample, with 0 indicating no reported music practice, this secondary exposure reflects overall weekly music exposure in the cohort rather than a purely within-musician measure of dose. These potential auditory and cognitive processes may help explain the observed associations between music participation and later language performance, although further work is needed to determine their relative contributions and to test them more directly.

### 4.2 Interpretation of global cortical morphometry in the music-language association

Global cortical measures provide summary indices of broad cortical organization and development. Our statistical mediation analyses indicated that global cortical surface area and volume, but not cortical thickness or sulcal depth, statistically accounted for part of the associations between sustained music participation and higher language scores. Because cortical volume reflects the combined contribution of surface area and thickness and is often more closely related to surface area than to thickness, the similarity of the surface area and volume findings in our analyses is not unexpected (Winkler et al., 2010). Prior studies that more closely align our findings suggest that language-related variation in youth may, in some contexts, be more evident in cortical surface area than in cortical thickness. For example, in children and adolescents aged 10–16 years, Bahar et al. (2024) reported surface-area differences in developmental language disorder without corresponding group differences in cortical thickness. Similarly, in 5- to 9-year-old children, Merz et al. (2020) reported that richer language input was associated with greater cortical surface area. In a related line of work, Seither-Preisler et al. (2014) followed 7- to 9-year-old children and found that larger auditory cortex size was associated with better musical, literacy, and attentional skills.

At the same time, other studies have emphasized cortical thickness rather than surface area, although they differ from the present study in both age window and analytic focus. In a longitudinal sample spanning late childhood through adolescence (ages 6–18 years), Hudziak et al. (2014) found not a simple overall association between music training and cortical thickness, but rather an age-by-training interaction, suggesting that musical training was related to the rate of cortical thickness maturation across development rather than to a stable thickness difference at a narrower late-childhood stage. In the language literature, thickness findings have likewise often been linked to much earlier developmental exposures: vocabulary growth during preschool (14–58 months) and parental language input before age 4 (18–42 months) have been associated with later cortical thickness or thickness change in language-related regions (Asaridou et al., 2017; Demir-Lira et al., 2021). Thus, studies emphasizing thickness often examine broader developmental change or the longer-term effects of language experience during the first years of life, rather than whether global cortical thickness measured in late childhood statistically accounts for the association between music participation and later language outcomes.

These differing patterns may reflect the fact that cortical surface area, thickness, and sulcal depth are most informative at different developmental stages and for different questions. Developmental studies indicate that thickness and surface area follow nonparallel trajectories across childhood and adolescence (Tamnes et al., 2017; Wierenga et al., 2014). Thickness tends to show more monotonic age-related decreases across much of development, whereas surface area follows a different trajectory. In addition, apparent cortical thinning during development may partly reflect increasing myelination and changing gray-white matter contrast rather than only stable anatomical differences (Natu et al., 2019). Sulcal depth, by contrast, reflects cortical folding geometry rather than cortical expansion per se and appears to capture a partly distinct morphometric dimension with demonstrable genetic influences (Pizzagalli et al., 2020; van der Meer et al., 2021). Moreover, prior links between sulcal depth and behavior have often been region-specific rather than global, including associations with verbal working memory (Yao et al., 2023). Accordingly, global sulcal depth in late childhood may be less sensitive to the kind of broad cortical variation captured by surface area and volume in the present study. Within this framework, cortical thickness may be especially sensitive to maturational remodeling in studies spanning wider age ranges or explicitly modeling developmental trajectories, whereas surface area and volume may provide relatively more stable indices of broad cortical variation in cohorts centered on late childhood. This may help explain why, in our study, global surface area and volume, but not thickness or sulcal depth, statistically accounted for the association between sustained music participation and later language outcomes.

The modest proportion of the music-language association statistically accounted for by global cortical measures (4 to 8%) suggests that these measures captured only a small fraction of the observed associations and that other cognitive, behavioral, educational, environmental, or genetic factors likely contribute to additional variation. Accordingly, global cortical morphometric measures should not be interpreted as individual-level predictive markers in this context. The modest attenuation observed after including additional prenatal/perinatal and behavioral variables, despite an otherwise unchanged overall pattern of findings, suggests that factors within these domains may explain part of the association. At the same time, this attenuation may also reflect overadjustment or collinearity among correlated measures of early environment and child functioning.

### 4.3 Implications of the findings

The observed association between sustained music participation and higher language performance from late childhood to early adolescence suggests that music participation may serve as one marker of developmental variation in language-related outcomes. Although the adjusted standardized differences in language outcomes between children with and without sustained music participation were modest (approximately 0.08 to 0.17 SD units), these effect sizes are consistent with contemporary perspectives emphasizing that small effects may still be meaningful in large-scale population-based and educational research (Kraft, 2020; Dick et al., 2021; Funder and Ozer, 2019). These differences have limited utility for individual-level prediction but may still carry population-level relevance over time, particularly across the transition from late childhood to early adolescence, a developmental period marked by substantial neural, cognitive, and educational change (Dahl et al., 2018).

These findings should nevertheless be interpreted in light of the baseline group differences, which suggest that music participation may function partly as a marker of broader family and extracurricular enrichment. Children with sustained music participation were more likely to come from higher-income and more highly educated households and to participate more frequently in other non-music extracurricular activities, indicating that, even after adjustment, the observed associations may reflect both music-related experiences related to music participation and broader environmental advantages that co-occur with sustained enrichment.

More broadly, prior work suggests that music experience may relate not only to auditory-language processing but also to cognitive processes relevant to learning, including attention and executive functioning (Besson et al., 2011; Moreno et al., 2011; Patel, 2011, 2012). Although the present study did not directly test these behavioral pathways, the observed global cortical associations may plausibly reflect broad neurodevelopmental correlates of both linguistic and non-linguistic aspects of learning. At the same time, brain structure is only one of many factors associated with cognitive performance and language development. In practical terms, these findings support continued investigation of music participation as a potentially relevant environmental correlate of language development in educational settings. Any such implications, however, should be interpreted primarily at the population level and considered within the broader context of individual differences, learning environments, and other interventions, given that language development is shaped by multiple biological, cognitive, and environmental factors.

### 4.4 Strengths and limitations

This study has several strengths. The longitudinal design, with music participation and brain structure measured at baseline and language outcomes assessed at 2-year follow-up, supports prospective interpretation of the observed exposure-outcome associations and helps reduce concerns about reverse causation for these associations. Importantly, adjustment for baseline language performance helps account for pre-existing differences in language ability and supports interpretation of the findings as prospective associations with follow-up language performance rather than simple cross-sectional associations. Furthermore, extensive adjustment for demographic, socioeconomic, prenatal/perinatal, behavioral, and extracurricular factors helped account for a wide range of measured confounders. Finally, the inclusion of sensitivity analyses in a twin subsample allowed us to examine whether broadly similar patterns were present in a subset with shared family background. Results were broadly similar across models with different levels of covariate adjustment, suggesting that the main findings were not entirely dependent on a single covariate specification. These design features support the consistency of the findings in this population-based longitudinal cohort.

Despite these strengths, several limitations should be acknowledged. First, as an observational study, definitive causal inferences cannot be made. In addition, because music participation and cortical measures were both assessed at baseline, temporal precedence of the candidate mediators cannot be established, and these analyses do not support causal mediation inference. The mediation results should therefore be interpreted as associational decomposition rather than evidence of a causal neurobiological pathway. Second, although our models adjusted for socioeconomic factors, baseline language performance, non-music extracurricular participation, behavioral measures, and study site, music participation may still reflect broader enrichment contexts not fully captured by the available covariates. Residual confounding by unmeasured familial, environmental, or individual factors cannot be entirely excluded. The twin sensitivity analysis allowed us to examine whether similar patterns were present in a subset with shared family background. Although the pattern for sustained music participation was directionally consistent with the full-sample findings, the analysis did not constitute a formal co-twin control design because the limited number of discordant twin pairs precluded within-pair inference. The results should therefore be interpreted as sensitivity findings rather than confirmatory evidence of causal effects. Third, the observed effect sizes were small, indicating that the findings have limited predictive value for individual children and should be interpreted primarily at the population level. Fourth, music participation measures were based on parent report and did not capture all dimensions of musical experience, such as type of musical activity, instructional context, or quality of instruction. At the same time, these measures likely capture typical real-world participation in music activities, which may enhance the ecological relevance of the findings. Future studies using more detailed assessments, such as activity logs, teacher reports, or repeated measures of musical engagement, may reduce measurement error and provide finer-grained information about which dimensions of music participation are most strongly associated with language outcomes. Fifth, the Crystallized Cognition Composite includes the Picture Vocabulary and Oral Reading Recognition measures, so these outcomes are related rather than fully independent and should be interpreted as complementary indices of language-related performance. Sixth, the mediation analyses focused on global cortical measures, which can serve as broad summary indices in population-level neurodevelopmental analyses but do not provide regional specificity regarding potential underlying neural mechanisms. Because music and language functions are supported by distributed but regionally specialized neural systems, whole-brain summary measures may miss or dilute more localized regional associations. Future studies using regionally specific cortical, subcortical, and white matter measures are needed to clarify regional neural correlates of music-language associations.

## 5 Conclusion

In this large, population-based longitudinal cohort, sustained music participation in late childhood was associated with modestly higher language performance 2 years later, and global cortical surface area and volume statistically accounted for a small proportion of these associations. These findings suggest that sustained music participation may relate to language development not only at the behavioral level but also through association with broader patterns of cortical structure during late childhood. The associations remained evident after adjustment for baseline language performance and multiple potential confounders, and patterns observed in the twin were directionally consistent with those in the full-sample results. Overall, this study provides longitudinal evidence that real-world music participation may be a relevant environmental correlate of language development, primarily at the population level, and highlights the need for further intervention-based and regionally specific neuroimaging studies to clarify underlying mechanisms.

## Data Availability

The data analyzed in this study were obtained from the Adolescent Brain Cognitive Development (ABCD) Study through the National Institute of Mental Health Data Archive. ABCD Study data are currently available to qualified researchers under controlled access through the NIH Brain Development Cohorts (NBDC) Data Hub. Access requires an approved Data Use Certification and compliance with applicable ABCD Study and NIH data use, training, and security requirements. Inquiries can be directed to the corresponding author.
