# Supplementary material for: Music participation, global cortical morphometry, and later language outcomes in the ABCD study
Source: Front Psychol. 2026 Aug 5;17:1852932. doi: 10.3389/fpsyg.2026.1852932 (PMC13485715; doi:10.3389/fpsyg.2026.1852932)
Supplement: Supplementary file 1 [file Supplementary_file_1.pdf]

eMethod 1. Baseline characteristics of participants with and without 2-year language data

eMethod 2. MRI acquisition, processing, quality control, and derivation of cortical measures

eFigure 1. Flowchart of twin subsample selection for sensitivity analyses.

eTable 1. Baseline music participation exposure variables derived from Parent-reported Sports and Activities Involvement Questionnaire (SAIQ).

eTable 2. Language outcomes at 2-year follow-up assessed using NIH Toolbox Cognition Battery.

eTable 3. Global cortical morphometric measures derived from high-resolution T1-weighted MRI using standardized preprocessing and surface-based morphometric pipelines.

eTable 4. Covariates included in the analyses, including baseline demographic, socioeconomic, prenatal/perinatal, behavioral, and enrichment variables.

eTable 5. Correlations of baseline characteristics with music practice intensity in the full sample (N = 5,993).

eTable 6. Expanded covariate-adjusted mediation analyses evaluating global cortical morphometric measures as statistical mediators of the associations between baseline music exposure variables and 2-year language outcomes.

eReferences

eMethod 1. Baseline characteristics of participants with and without 2-year language data

A subset of participants (N = 6,571) with available 2-year language data in the ABCD 3.0 data release was identified. To assess potential selection bias, we compared baseline characteristics, including age, sex, race/ethnicity, annual household income, parents' marital status and education, preterm birth status, maternal alcohol and tobacco use during pregnancy, ADHD DSM-5 scale, social problem score, internalizing and externalizing scores, and counts of broader enrichment activities, between participants with and without 2-year language data.[1] We applied a chi-square automatic interaction detection (CHAID) analysis to test whether these characteristics could reliably distinguish participants with versus without 2-year language follow-up.[2] he full sample (N = 11,878) was randomly divided, stratified by follow-up status, into a 70% training set and a 30% validation set. The CHAID model achieved a classification accuracy of 56.2% on the validation set, virtually identical to the chance level of 55.3%, defined by the proportion of participants with 2-year language data. This difference of less than 1 percentage point indicates that measured baseline characteristics did not meaningfully differentiate participants with versus without 2-year language follow-up. Accordingly, these results provide limited evidence that exclusion of participants with missing language data introduced substantial selection bias in the analytic sample.[3]

eMethod 2. MRI acquisition, processing, quality control, and derivation of cortical measures

Complete MRI acquisition parameters and image processing pipelines are described elsewhere.[4,5] Key procedures are summarized below:

- MRI Acquisition:
  Structural MRI data were acquired at 21 sites on 3-T Siemens, GE, and Philips scanners using a standardized protocol developed by the ABCD Imaging Acquisition Workgroup. Each participant underwent high-resolution 3D T1-weighted and T2-weighted imaging.

- Automated Processing:
  The ABCD Data Analysis, Informatics, and Resource Center (DAIRC) conducted centralized automated processing of all scans. Preprocessing included gradient nonlinearity correction, intensity inhomogeneity correction, and registration to a standard reference space. Cortical surface reconstruction and subcortical segmentation were performed using FreeSurfer.

- Quality Control:
  Trained DAIRC technicians manually reviewed all processed data. Quality control procedures included assessment of motion artifacts, intensity inhomogeneity, white matter underestimation, pial overestimation, and magnetic susceptibility artifacts.

- Derivation of Cortical Metrics:
  Global cortical measures were derived from T1-weighted structural MRI data processed using standardized ABCD Study pipelines. Cortical surface reconstruction used intensity- and topology-based segmentation to delineate the gray-white and pial surfaces. Total cortical surface area was defined as the sum of vertex-level surface areas, total cortical volume as the gray matter volume between the two surfaces, mean cortical thickness as the average distance between the gray-white and pial surfaces, and mean sulcal depth as the average depth of cortical folds relative to a spherical atlas.

eFigure 1. Flowchart of twin subsample selection for sensitivity analyses.

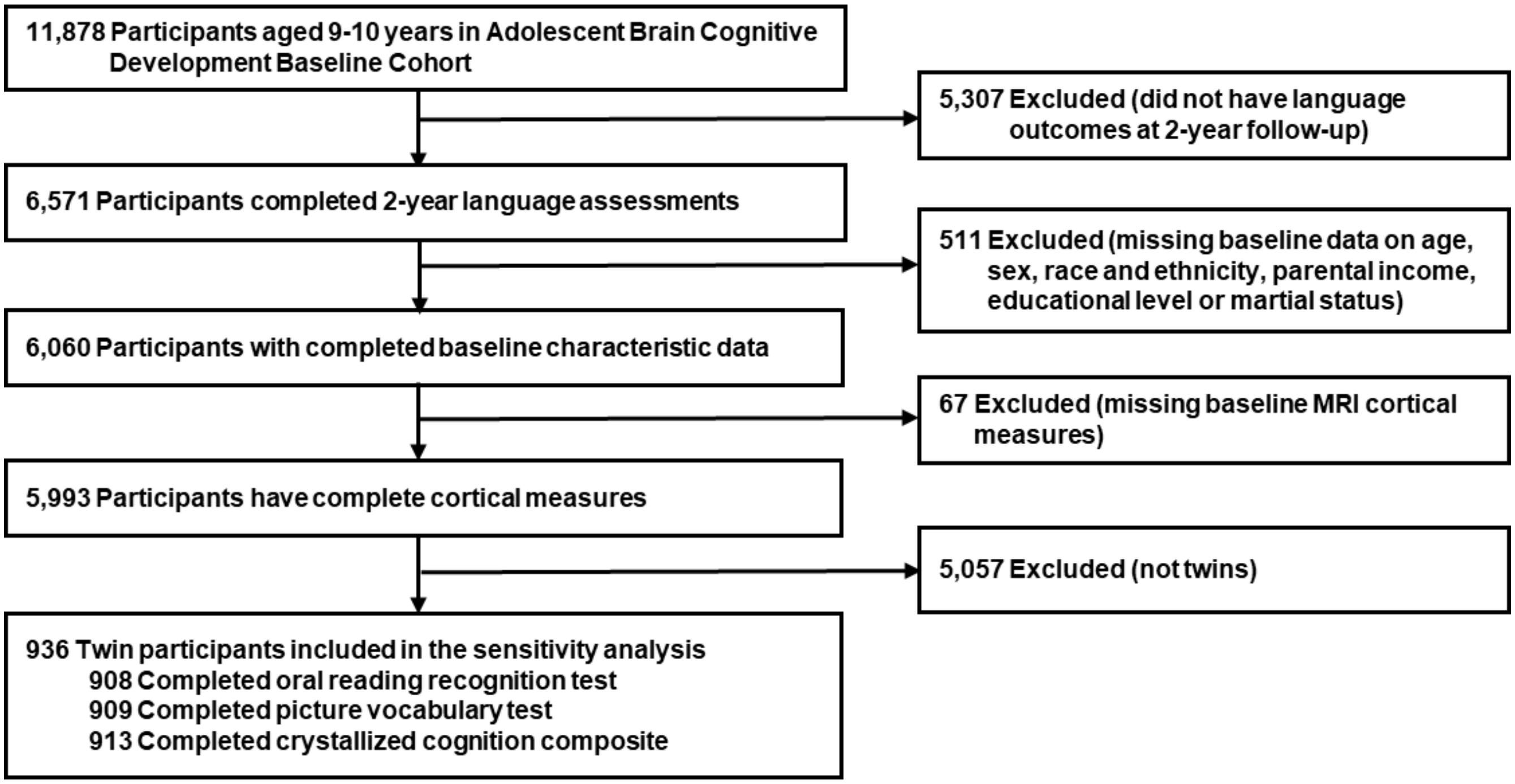

eTable 1. Baseline music participation exposure variables derived from Parent-reported Sports and Activities Involvement Questionnaire (SAIQ).

**Description**	**ABCD Variable Name**	**Original coding**	**Coding for Analysis**
Sustained music participation: whether the child has ever participated in music activities (singing, choir, guitar, piano, drums, violin, glute, band, rock band, orchestra) for ≥4 continuous months	sai_p_activities___23	0 = No; 1 = Yes	Binary indicator: 0 = No; 1 = Yes
Practice frequency (days per week)	sai_p_music_perwk	1 = 1; 2 = 2; 3 = 3; 4 = 4; 5 = 5; 6 = 6; 7 = 7; 8 = Once every 2 weeks	Codes 1–7 were converted to corresponding days/week values (1–7); code 8 converted to 0.5 days/week
Practice duration (minutes per session)	sai_p_music_tspent	1 = less than 30 minutes; 2 = 30; 3 = 45; 4 = 60 (1 hr); 5 = 90 (1.5 hrs) 90; 6 = 120 (2 hrs); 7 = 150 (2.5 hrs); 8 = 180 (3 hrs); 9 = greater than 3 hours	Codes 1–2 were recoded as 30 min; codes 3–4 as 60 min; code 5 as 90 min; code 6 as 120 min; code 7 as 150 min; and codes 8–9 as 180+ min

Primary exposure: sustained music participation, a binary variable defined as participation in organized music activities (e.g., singing, choir, guitar, piano, drums, violin, flute, band, rock band, orchestra) for ≥4 continuous months (yes/no). Secondary exposure: practice intensity, calculated as minutes per week (days/week × minutes/session), assuming one practice session per day. This approach is consistent with the structure of the SAIQ, which records activity frequency and typical session duration separately but does not query multiple sessions per day. In the full sample, practice intensity was modeled continuously, with 0 indicating no reported music practice, and was standardized to a z score before analysis; coefficients therefore represent the association per 1-standard deviation increase in weekly practice intensity.

eTable 2. Language outcomes at 2-year follow-up assessed using NIH Toolbox Cognition Battery.

Description	ABCD Variable Name	Original coding	Coding for Analysis
Crystallized Cognition Composite: composite score derived from the Picture Vocabulary and Oral Reading Recognition tests, reflecting accumulated knowledge and language-based skills	nihtbx_cryst_agecorrected	Age-adjusted standardized NIH Toolbox scores (mean = 100, SD = 15); higher scores indicate better performance	Used as continuous variables without recoding
Picture Vocabulary Test: assesses receptive vocabulary and verbal comprehension	nihtbx_picvocab_agecorrected		
Oral Reading Recognition Test: assesses reading decoding and word recognition accuracy	nihtbx_reading_agecorrected		

eTable 3. Global cortical morphometric measures derived from high-resolution T1-weighted MRI using standardized preprocessing and surface-based morphometric pipelines.

Description	ABCD Variable Name	Original coding	Coding for Analysis
Mean cortical thickness	smri_thick_cdk_mean	Continuous values in mm	Used as continuous variables without recoding
Mean sulcal depth	smri_sulc_cdk_mean	Continuous values in mm	
Total cortical surface area	smri_area_cdk_total	Continuous values in mm^2	
Total cortical volume	smri_vol_cdk_total	Continuous values in mm^3	

eTable 4. Covariates included in the analyses, including baseline demographic, socioeconomic, prenatal/perinatal, behavioral, and enrichment variables.

**Category**	**ABCD variable Name**	**Original coding**	**Coding for Analysis**
**Demographic factors**			
Age at interview	interview_age	Continuous, in months	Continuous, converted to years
Sex assigned at birth	sex	1 = Male; 2 = Female; other options were available in the questionnaire but only 1 and 2 were present in the released baseline dataset	Same as original coding
Race/ethnicity	race_ethnicity	1 = White; 2 = Black; 3 = Hispanic; 4 = Asian; 5 = Other	Same as original coding
**Socioeconomic factors**			
Annual household income, $<br>What is your TOTAL COMBINED FAMILY INCOME for the past 12 months? This should include income (before taxes and deductions) from all sources, wages, rent from properties, social security, disability and/or veteran's benefits, unemployment benefits, workman's compensation, help from relative (include child payments and alimony)	demo_comb_income_v2	1= Less than $5,000; 2=$5,000 through $11,999; 3=$12,000 through $15,999; 4=$16,000 through $24,999; 5=$25,000 through $34,999; 6=$35,000 through $49,999; 7=$50,000 through $74,999; 8= $75,000 through $99,999; 9=$100,000 through $199,999; 10=$200,000 and greater. 999 = Don't know; 777 = Refuse to answer	Binary:<br>< $50,000 (codes 1-6);<br>≥ $50,000 (codes 7-10)
Parents’ marital status<br>Are you now married, widowed, divorced, separated, never married or living with a partner?	demo_prnt_marital_v2	1 = Married; 2 = Widowed; 3 = Divorced; 4 = Separated; 5 = Never married; 6 = Living with partner; 777 = Refused to answer	3-class:<br>Married (code 1); Single Parent (codes 2-5); Living with Partner (code 6)
Parents’ education<br>What is the highest grade or level of school you have completed or the highest degree you have received?	Demo_prnt_ed_v2	0 = Never attended/Kindergarten only; 1 = 1st grade; 2 = 2nd grade; 3 = 3rd grade; 4 = 4th grade; 5 = 5th grade; 6 = 6th grade; 7 = 7th grade; 8 = 8th grade; 9 = 9th grade; 10 = 10th grade; 11 = 11th grade; 12 = 12th grade; 13 = High school graduate; 14 = GED or equivalent Diploma General; 15 = Some college; 16 = Associate	Binary<br>< Some college (codes 0-14);<br>≥ Some college (codes 15-21)

		degree: Occupational; 17 = Associate degree: Academic Program; 18 = Bachelor's degree (ex. BA; 19 = Master's degree (ex. MA; 20 = Professional School degree (ex. MD; 21 = Doctoral degree (ex. PhD; 777 = Refused to answer	
**Perinatal and prenatal factors**			
Preterm-birth status<br>Was the child born prematurely?	devhx_12a_p	1 = Yes; 0 = No; 999 = Don't know	Three-class: Yes, No, Don't know
Maternal alcohol use in pregnancy	devhx_9_alcohol	1 = Yes; 0 = No; 999 = Don't know	
Maternal tobacco use in pregnancy	devhx_9_tobacco	1 = Yes; 0 = No; 999 = Don't know	
**Child behavior and emotional functioning assessed using Child Behavior Checklist (CBCL) T scores**			
ADHD DSM-5 Scale	cbcl_scr_dsm5_adhd_t	Continuous T-scores (range 0-100; mean = 50, SD = 10)	Continuous T-scores
Social Problems	cbcl_scr_syn_social_t		
Internalizing symptoms	cbcl_scr_syn_internal_t		
Externalizing symptoms	cbcl_scr_syn_external_t		
**Broader enrichment activities from Parent-reported Sports and Activities Involvement Questionnaire (SAIQ), capturing participation in the following activity items for ≥4 continuous months**			
Team Sports (count: baseball/softball, basketball, football, soccer)	sai_p_activities___1<br>sai_p_activities___2<br>sai_p_activities___5<br>sai_p_activities___15	Binary participation: 1= Yes; 0= No	Integer: total count of endorsed items
Individual Sports (count: gymnastics, martial arts, swimming)	sai_p_activities___6<br>sai_p_activities___10<br>sai_p_activities___17	Binary participation: 1= Yes; 0= No	Integer: total count of endorsed items
Creative arts (count: ballet, dance, drawing, painting, graphic art, photography, pottery, sculpting)	sai_p_activities___0<br>sai_p_activities___24	Binary participation: 1= Yes; 0= No	Integer: total count of endorsed items

Note: Non-music extracurricular activities were defined as the total count of team sports, individual sports, and creative arts activity items endorsed for ≥4 continuous months in the Sports and Activities Involvement Questionnaire.

eTable 5. Correlations of baseline characteristics with music practice intensity in the full sample (N = 5,993).

Characteristics	Mean (SD) or No. (%)	Correlation with music practice intensity (95% CI)	P value
**Demographics**			
Age, y	10.0 (0.6)	0.08 (0.06, 0.11)	<.001
Sex, female	2,823 (47.1)	0.04 (0.01, 0.06)	.006
Race and ethnicity, white	3,530 (58.9)	0.01 (-0.02, 0.04)	.42
**Socioeconomic factors**			
Household income ≥$50,000	4,399 (73.4)	0.05 (0.02, 0.07)	<.001
Parental marital status, married	4,296 (71.7)	0.04 (0.01, 0.06)	.006
Parental education, ≥ some college	5,236 (87.4)	0.05 (0.02, 0.07)	<.001
**Perinatal and prenatal factors**			
Preterm-birth status	1,191 (19.9)	-0.05 (-0.07, -0.02)	<.001
Maternal alcohol use during pregnancy	178 (3.0)	0.02 (-0.01, 0.05)	.11
Maternal tobacco use during pregnancy	251 (4.2)	-0.02 (-0.04, 0.01)	.19
**Child behavioral and emotional functioning[a]**			
ADHD DSM-5 Scale	53.1 (5.6)	-0.03 (-0.06, -0.01)	.02
Social problems	52.7 (4.6)	-0.01 (-0.04, 0.02)	.46
Internalizing symptoms	48.6 (10.5)	-0.01 (-0.03, 0.02)	.67
Externalizing symptoms	45.5 (10.2)	-0.02 (-0.05, 0.00)	.08
**Broader enrichment activities**			
Non-music activities[b]	2.83 (2.01)	0.10 (0.08, 0.13)	<0.001

Music practice intensity was defined as weekly minutes of music practice. In the full sample, children without music participation were coded as 0 min/week. Values are Pearson correlation coefficients with 95% confidence intervals. For binary variables, correlations were computed using 0/1 coding (equivalent to a point-biserial correlation). For categorical variables with more than two levels, one-versus-rest coding was applied. Confidence intervals were calculated using standard errors.

[a] ADHD, Social Problems, Internalizing symptoms, and Externalizing symptoms were assessed using Child Behavior Checklist (CBCL) T-scores, standardized to a mean of 50 (SD, 10).

[b] Non-music activities were defined as the total count of non-music extracurricular activity items, including team sports, individual sports, and creative arts, in which the child participated for ≥4 continuous months, as reported in the Sports and Activities Involvement Questionnaire.

eTable 6. Expanded covariate-adjusted mediation analyses evaluating global cortical morphometric measures as statistical mediators of the associations between baseline music exposure variables and 2-year language outcomes.

Panel A. Sustained Music Participation

Language Outcome	Cortical Mediator	Total Effect, β_T (95% CI)	Indirect Effect β_I (95% CI)	q value	Direct Effect β_D (95% CI)	Proportion mediated, %
Crystallized Cognition Composite (N = 5,818)	Thickness	2.066 (1.378, 2.754)	0.001 (-0.019, 0.022)	0.909	2.064 (1.377, 2.752)	—
	Sulcal Depth		-0.001 (-0.016, 0.011)	0.909	2.066 (1.378, 2.754)	—
	Surface Area		0.101 (0.029, 0.182)	0.009	2.008 (1.324, 2.692)	4.9
	Volume		0.106 (0.030, 0.191)	0.009	2.003 (1.32, 2.687)	5.1
Picture Vocabulary Test (N = 5,735)	Thickness	2.465 (1.712, 3.218)	0.000 (-0.012, 0.014)	0.911	2.464 (1.711, 3.217)	—
	Sulcal Depth		-0.002 (-0.024, 0.016)	0.911	2.465 (1.712, 3.218)	—
	Surface Area		0.098 (0.028, 0.179)	0.010	2.410 (1.66, 3.159)	4.0
	Volume		0.093 (0.026, 0.172)	0.010	2.412 (1.662, 3.162)	3.8
Oral Reading Recognition Test (N = 5,723)	Thickness	1.190 (0.492, 1.888)	0.001 (-0.026, 0.03)	0.909	1.188 (0.49, 1.886)	—
	Sulcal Depth		0.000 (-0.010, 0.012)	0.909	1.191 (0.493, 1.889)	—
	Surface Area		0.076 (0.021, 0.143)	0.012	1.146 (0.451, 1.842)	6.4
	Volume		0.091 (0.026, 0.167)	0.012	1.137 (0.442, 1.832)	7.6

Panel B. Practice Intensity

Language Outcome	Cortical Mediator	Total Effect β (95% CI)	Indirect Effect β (95% CI)	q value	Direct Effect β (95% CI)	Proportion mediated, %
Crystallized Cognition Composite (N = 5,818	Thickness	0.669 (0.361, 0.977)	0.000 (-0.009, 0.01)	0.950	0.669 (0.361, 0.977)	—
	Sulcal Depth		0.0 (-0.005, 0.007)	0.950	0.668 (0.360, 0.976)	—
	Surface Area		0.036 (0.004, 0.072)	0.035	0.642 (0.336, 0.949)	5.4
	Volume		0.040 (0.006, 0.078)	0.035	0.643 (0.337, 0.949)	6.0
Picture Vocabulary Test (N = 5,735)	Thickness	0.841 (0.500, 1.182)	0.000 (-0.006, 0.006)	0.950	0.841 (0.500, 1.182)	—
	Sulcal Depth		0.001 (-0.008, 0.01)	0.950	0.840 (0.499, 1.181)	—
	Surface Area		0.035 (0.004, 0.071)	0.037	0.819 (0.480, 1.158)	4.2
	Volume		0.035 (0.005, 0.07)	0.037	0.819 (0.479, 1.158)	4.2
Oral Reading Recognition Test (N = 5,723)	Thickness	0.279 (-0.038, 0.595)	0.000 (-0.012, 0.013)	0.950	0.279 (-0.038, 0.595)	—
	Sulcal Depth		0.000 (-0.005, 0.005)	0.950	0.279 (-0.038, 0.596)	—
	Surface Area		0.027 (0.003, 0.056)	0.040	0.263 (-0.053, 0.578)	—
	Volume		0.034 (0.005, 0.067)	0.040	0.260 (-0.055, 0.575)	—

Expanded covariate-adjusted mediation analyses included adjustment for baseline language performance; demographic factors (age, sex, race and ethnicity); socioeconomic factors (household income, parental education, and marital status); prenatal/perinatal factors (preterm birth status, maternal alcohol use during pregnancy, and maternal tobacco use during pregnancy); child behavioral and emotional functioning (CBCL ADHD T-score, Social Problems, Internalizing symptoms, and Externalizing symptoms); number of non-music extracurricular activities (sports and creative arts); and study site. β_T denotes the total effect, β_D denotes the direct effect adjusted for the mediator, and β_I denotes the indirect effect. The indirect effect was estimated using the product-of-coefficients method; proportion mediated was calculated as the indirect effect divided by the total effect. Ninety-five percent confidence intervals for indirect effects were estimated using Monte Carlo simulation, whereas confidence intervals for total and direct effects were calculated using Wald-type methods. q values represent Benjamini-Hochberg false discovery rate–adjusted p values for the indirect effects; $q < .05$ was considered statistically significant. The total effect reflects the association between the exposure and outcome before inclusion of the mediator and is therefore identical across mediator-specific models. "—" indicates that the proportion mediated was not estimated because either the indirect effect or the total effect was not statistically significant.

eReferences

1. Howe CJ, Cole SR, Lau B, Napravnik S, Eron JJ, Jr. Selection Bias Due to Loss to Follow Up in Cohort Studies. *Epidemiology*. Jan 2016;27(1):91–7. doi:10.1097/ede.0000000000000409
2. Venkatasubramaniam A, Wolfson J, Mitchell N, Barnes T, JaKa M, French S. Decision trees in epidemiological research. *Emerg Themes Epidemiol*. 2017;14:11. doi:10.1186/s12982-017-0064-4
3. Barkho W, Carnes NC, Kolaja CA, et al. Utilizing machine learning to predict participant response to follow-up health surveys in the Millennium Cohort Study. *Sci Rep*. Oct 28 2024;14(1):25764. doi:10.1038/s41598-024-77563-8
4. Casey BJ, Cannonier T, Conley MI, et al. The Adolescent Brain Cognitive Development (ABCD) study: Imaging acquisition across 21 sites. *Dev Cogn Neurosci*. Aug 2018;32:43–54. doi:10.1016/j.dcn.2018.03.001
5. Hagler DJ, Jr., Hatton S, Cornejo MD, et al. Image processing and analysis methods for the Adolescent Brain Cognitive Development Study. *Neuroimage*. Nov 15 2019;202:116091. doi:10.1016/j.neuroimage.2019.116091
